# Pure alpha-fetoprotein-producing neuroendocrine carcinoma of the pancreas: a case report

**DOI:** 10.1186/s12876-015-0246-x

**Published:** 2015-02-12

**Authors:** Xiang Zhu, Huijuan Yong, Li Zhang, Yonghui Huang, Jie Zheng, Cuiling Liu, Dianrong Xiu, Pan Zhang

**Affiliations:** 1 Department of Pathology, Peking University Third Hospital, Beijing, 100191 China; 2 Department of Pathology, Peking University Health Science Center, Beijing, 100191 China; 3 Department of Gastroenterology, Peking University Third Hospital, Beijing, 100191 China; 4 Department of Surgery, Peking University Third Hospital, Beijing, 100191 China

**Keywords:** Alpha-fetoprotein, Neuroendocrine carcinoma, Pancreas

## Abstract

**Background:**

Alpha-fetoprotein (AFP)-producing pancreatic neuroendocrine tumors (pNETs) are rare, and the few reported cases usually coexisted with other malignant components such as adenocarcinoma or hepatoid carcinoma. We present here the first case of pure AFP-producing pNET.

**Case presentation:**

A 56-year-old male patient underwent resection of the pancreatic tail and body because of elevated serum AFP levels and pancreatic mass. Microscopy showed that the tumor tissue consisted of large and small solid nests of polygonal cells. The tumor cells were positive for chromogranin A, synaptophysin, CD99, cytokeratin 19, pan-cytokeratin and β-catenin, and also showed diffuse immunoreactivity for AFP and human chorionic gonadotrophin. The mitotic rate was nearly 30 per 10 high-power fields and the Ki-67 index was nearly 25%. The histopathologic findings supported the diagnosis of an AFP-producing pNET. Other malignant components were not found. Serum AFP levels decreased to near-normal after operation and gradually increased to >1000 ng/ml at 5 months post-surgery. Recurrence and hepatic metastases were revealed by computed tomography. The patient died 21 months after surgery.

**Conclusion:**

This was the first case of pure AFP-producing pNET to be reported in the English literature. Serum AFP levels may provide useful information for monitoring the therapeutic effectiveness, early recurrence or metastases.

## Background

Pancreatic neuroendocrine tumors (pNETs) are rare malignant tumors of the pancreas and account for approximately 1-2% of all pancreatic neoplasms [1,2]. The origin of pNETs is not completely understood, but these tumors may arise from pluripotent stem cells within the exocrine pancreas [3]. pNETs may be divided into functional and nonfunctional tumors according to whether or not there is an associated clinical syndrome caused by the release of biologically active peptides. Because there are no specific clinical symptoms associated with nonfunctional pNETs, these neoplasms are frequently found incidentally and diagnosed at late stages [4,5].

Alpha-fetoprotein (AFP) has long been used as a tumor marker for hepatocellular carcinoma (HCC) and embryonic cell tumors. Elevated serum levels of AFP were also found in patients with carcinoma metastasis to the liver or non-neoplastic liver injury. Several cases of pNETs with elevated serum AFP levels have been reported, but most of these cases had liver metastasis at the time of diagnosis [6-8]. Rare cases of AFP-producing pNETs have been described in the English literature [9-14], but these tumors usually coexisted with other malignant components such as adenocarcinoma or hepatoid carcinoma. Here, we present the first case of pure AFP-producing pNET, in which the AFP-producing site was immunohistochemically confirmed in the tumor tissues. The clinico-pathological characteristics of this tumor were evaluated, and the literature about AFP-producing pNETs was reviewed.

## Case presentation

### Clinical course

A 56-year-old man was admitted to the Peking University Third Hospital in December of 2011 because of abnormal imaging of the pancreas and high serum AFP levels found during routine health checkup. He had no symptoms of hypoglycemia, diarrhea or abdominal pain. Fifteen months earlier, he suffered from a liver abscess with normal serum AFP, which was treated successfully with antibiotics. Cholecystolithiasis was diagnosed at that time. One month earlier, he had undergone laparoscopic cholecystectomy for gallstones at another hospital. Enlargement of the tail and body of the pancreas was demonstrated by computed tomography (CT), and a diagnosis of pancreatitis was considered. The patient did not smoke but drank alcohol occasionally. Physical examination on admission revealed no specific findings.

Laboratory tests showed that serum AFP levels were elevated to 321.4 ng/ml (normal: 0–20 ng/ml). The levels of other tumor markers (carcinoembryonic antigen [CEA], carbohydrate antigen 19–9 [CA199], carbohydrate antigen 125, and prostatic antigens) were all within normal limits. There was no serologic evidence of hepatitis B or C. Blood cell counts, erythrocyte sedimentation rate, and coagulation tests were normal. Levels of serum aminotransferases, alkaline phosphatase, gamma-glutamyl transferase, albumin, urea nitrogen, creatinine, amylase, lipase, glucose, human chorionic gonadotrophin (HCG), and immunoglobulins were all normal. Testicular ultrasonography revealed a cystic mass in the left epididymidis.

**Figure 1:**
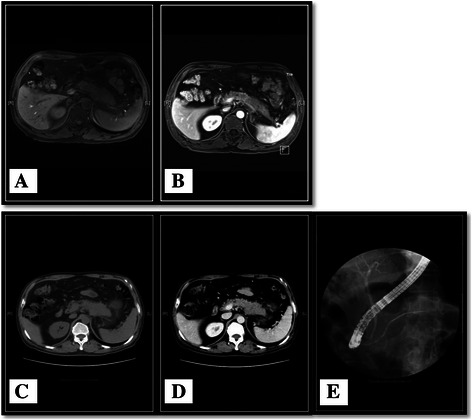
**Imaging examinations.** MRI showed enlargement of the pancreas body and tail with poor enhancement after gadolinium injection. Some enlarged peripancreatic lymph nodes were noted **(A, B)**. Abdominal CT revealed diffuse enlargement of the pancreas body and tail, which appeared to be hypoattenuated with an unclear margin. A mass measuring 5.2 × 4.8 × 4.1 cm with probable encasement of the splenic vein was observed in the enlarged pancreas on contrast-enhanced CT **(C, D)**. None of the imaging examinations revealed abnormal findings in the liver. The main pancreatic duct, common bile duct, and intrahepatic bile ducts were normal on endoscopic retrograde cholangiopancreatography **(E)**.

Imaging examinations are shown in Figure 1. Abdominal CT revealed diffuse enlargement of the body and tail of the pancreas, which appeared as a region of low-attenuation with an indistinct margin. A mass measuring 5.2 × 4.8 × 4.1 cm showing probable encasement of the splenic vein was found in the enlarged pancreas by contrast-enhanced CT. Magnetic resonance imaging confirmed enlargement of the body and tail of the pancreas with poor enhancement after gadolinium administration. Some enlarged peripancreatic lymph nodes were also noted. None of the imaging examinations showed abnormal findings in the liver. The main pancreatic duct, common bile duct, and intrahepatic bile ducts were normal on endoscopic retrograde cholangiopancreatography. The patient underwent a resection of the tail and body of the pancreas, splenectomy, and resection of four regional lymph nodes in January 2012. No metastatic neoplasm was found on the liver surface or in lymph nodes during surgery.

### Histopathological findings

**Figure 2:**
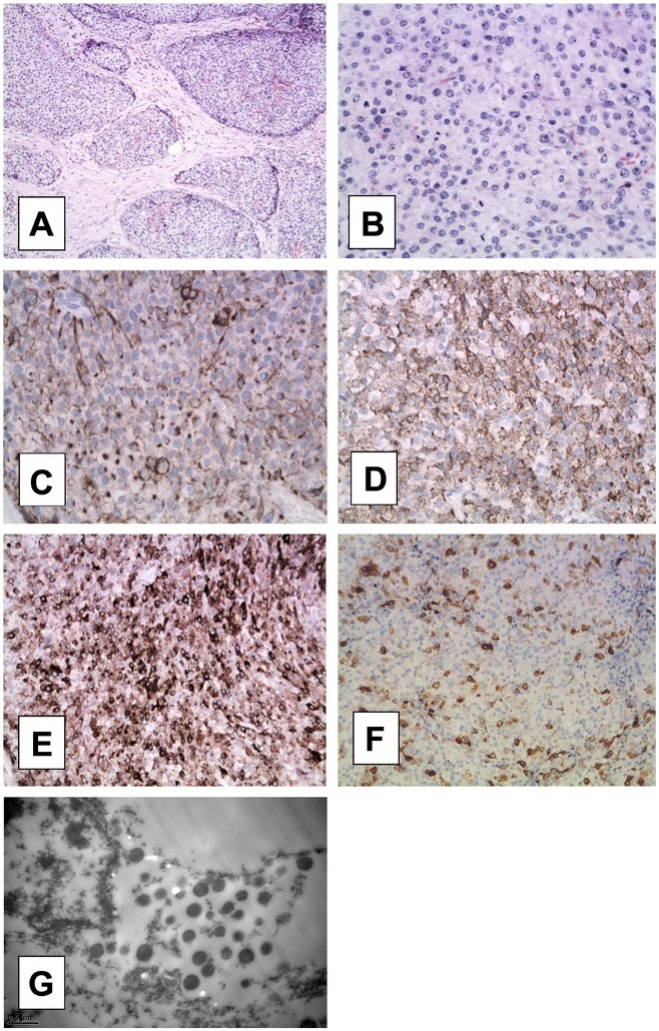
**Histopathological findings.** The tumor was predominantly composed of circumscribed cellular islands (**A**, 100×) comprised of large and small solid nests of polygonal cells that had moderate amounts of cytoplasm, and round to oval nuclei with minimal to moderate atypia (**B**, 400×). The mitotic cells were easy to find. The tumor cells widely expressed pan-cytokeratin, which appeared as typical dot-like positive on the nuclear side (**C**, 400×). CgA expression was patchy (**D**, 400×). Tumor cells exhibited partial strong AFP immunoreactivity with a granular pattern (**E**, 400×). Tumor cells focally showed strong HCG immunoreactivity (**F**, 400×). Transmission electron microscopic examination showed that the tumor cells contained electron-dense neuroendocrine granules measuring 230 nm in diameter **(G)**.

Grossly, a grey ill-define nodular mass, measuring 5 × 4.5 × 3.5 cm, was shown at the body and tail of the pancreas (Figure 2). Necrosis, hemorrhage and parapancreatic adipose tissues invasion were observed. Light microscopy revealed that the tumor tissue consisted of large and small solid nests of polygonal cells with a scattering of pancreas islets among them. No residual pancreatic tissue was found within the tumor. The tumor cells had moderate amounts, granular and eosinophilic cytoplasm, and round to oval nuclei with mild to moderate atypia. One or two red, moderately sized nucleoli were noted in some tumor cells. The mitotic rate was nearly 30 per 10 high-power fields. There was no differentiated component characteristic of adenocarcinoma, acinar cell carcinoma or hepatoid carcinoma of the pancreas. The surgical margins were negative for neoplastic infiltration. There was no evidence of vascular or perineural invasion. No lymph node metastasis was shown. Transmission electron microscopy showed that the tumor cells contained electron-dense neuroendocrine granules measuring 230 nm in diameter.

Immunohistochemistry showed patchy expression of chromogranin A (CgA) and synaptophysin (Syn). Tumor cells also showed diffuse immunoreactivity for AFP and focal strong immunoreactivity for hCG. Dot-like pan-cytokeratin (CKpan) reactivity near the nucleus, β-catenin reactivity at the cell membrane, diffuse CD99 expression in cytoplasm, and wide CK19 expression in tumor cells were observed. There was no expression of HapPar-1, CD10, CD56, or progesterone receptor (PR) in tumor cells. Ki-67 index was nearly 25%. The histopathology findings supported the diagnosis of a pure AFP-producing neuroendocrine carcinoma according to the forth edition of WHO Classification of neuroendocrine tumors [15].

### Follow-up

Serum AFP levels decreased to 23.15 ng/ml on day 29 post-surgery, but increased to 180.9 ng/ml on day 80. Lymph nodes metastasis and a recurrent mass in the residual pancreas were detected by CT. The patient was treated with a somatostatin (SS) analog (Sandostatin LAR) for 4 months. Serum AFP levels gradually increased to 1200 ng/ml 5 months after surgery, and liver metastases were found on CT. Hepatic metastases were treated with hepatic artery embolization and percutaneous radiofrequency ablation (RFA) in another hospital, and the serum AFP levels fluctuated around 700 ng/ml. His clinical condition gradually worsened, and he died 21 months after the original surgery. Other tumor markers (such as CEA and CA19-9) remained within the normal range during follow-up.

## Discussion

AFP is a tumor marker for HCC and had been used in the clinical practice for a long time. Because there is a large population of patients with hepatitis B or C virus infection in China, serum levels of AFP are measured routinely during health checkups to screen for HCC. The high levels of AFP in this patient originally raised the question as to whether there was a tumor or another condition causing the expression of AFP.

Pancreatic neuroendocrine neoplasms are rare, sporadic pancreatic tumors. Many pNETs are diagnosed at late stages because of the lack of symptoms in the case of non-functional tumors. The apparent incidence of pNETs had increased in recent years, likely due to improved detection methods, but it is still difficult to diagnosis pNETs at an early stage when the tumor do not invade adjacent tissues or produce distant metastases [1,2]. Histologically, pNETs consist of relatively homogeneous, small, round cells with uniform nuclei and cytoplasm. The expression of CgA is widely used to identify gastrointestinal NETs [16].

Only 23 cases of pNETs with elevated serum AFP levels have been described in the English literatures, and liver metastases were confirmed in most of these patients [6-14]. Among these cases, the AFP-producing sites were identified by immunohistochemical examination of the resected tumor in 7 cases only (see Table 1, including the current case). Interestingly, the presence of another malignant component such as adenocarcinoma (1 case) or hepatoid carcinoma (5 cases) coexisted with the neuroendocrine neoplasm in all these 7 cases except the present case. In our case, there were no histopathological features and patterns of immunoreactivity typically usually seen in carcinoma arising from pancreatic ducts, acinar cell, or hepatoid carcinoma. To the best of our knowledge, this is the first reported case of histologically-confirmed pure AFP-producing pNET without another coexisting malignant component.

**Table 1:** **Studies of AFP-producing pNETs or AFP-producing pancreatic tumors with a neuroendocrine component confirmed by immunohistochemistry**

Study	Year of publication	Age/sex	Location	Other components	Metastasis to the liver	Follow-up (months)/outcome
Paner et al. [9]	2000	57/M	Tail	Hepatoid	Yes	102/DOD
Lam et al. [10]	2001	64/F	Tail	Hepatoid	Yes	22/DOD
Oh et al. [11]	2006	21/M	Head	Hepatoid	NA	>7
Hameed et al. [12]	2007	41/F	Head	Hepatoid	No	27/DOD
Brandi et al. [13]	2008	68/F	Tail/body	Adenocarcinoma	Yes	12/DOD
Jung et al. [14]	2010	46/M	Head	Hepatoid	No	>4
Present case	NA	56/M	Tail/body	None	Yes	21/DOD

DOD: dead of disease; NA: not available.

In the present case, tumor cells exhibited dot-like CKpan reactivity near the nucleus, which is the classical CK expression pattern in neuroendocrine tumors. CD56 is usually expressed in pNETs [17], but was negative in this case. In the present case, tumor cells were positive for β-catenin at the cell membrane, not in the nucleus. Cells were diffusely positive for CD99 in the cytoplasm, but dot-like immunoreactivity was not observed near the nucleus. CD10 and PR were both negative. These findings suggested that the tumor was not a solid-pseudopapillary neoplasm of the pancreas. Previous studies described elevated serum HCG levels in patients with pNETs [8,18]. In the present case, serum HCG levels were normal, but immunohistochemisrty for HCG was positive in the resected tumor tissues.

Surgery is the only treatment that has the potential to cure patients with pNETs. Although the present patient was treated with radical surgery that achieved free resection margins and that did not exhibit regional lymph node metastases, the tumor relapsed and metastasized within months after surgery. More than 90% of pNETs express SS receptors, and biotherapy with long-acting SS analogs had been shown to slow tumor growth and improve survival in patients with pNETs [19,20]. However, recurrence and liver metastases were noted during the course of SS analogs treatment in the present case, suggesting that SS analogs are not always effective in preventing tumor growth or metastasis. Treatments with arterial embolization and percutaneous RFA for hepatic metastases may prolong the patient’s life.

Pancreatic neuroendocrine neoplasms include well-differentiated (low- to intermediate-grade) neuroendocrine tumors and poorly differentiated (high-grade) neuroendocrine carcinomas (NECs). NECs are defined by the presence of >20 mitoses per 10 high-power fields (and/or, >20% Ki67 index) according to the WHO Classification of neuroendocrine neoplasms of the pancreas [15]. On the basis of morphological criteria and the assessment of proliferation fraction, the grade of present case was NEC. According to the TNM classification system, the present case was Stage IIA (T3N0M0) at diagnosis. The grade (based on the proliferation rate) and stage (extent of disease) of this tumor were coincident.

In an analysis of the Surveillance Epidemiology and End Results (SEER) database, the 5-year survival of patients with poorly differentiated tumors was <38% for local disease, 21% for loco-regional disease, and <4% for metastatic disease, and the median survival for distant metastatic disease was only 5 months in patients with poorly differentiated carcinoma [21]. Some histological features such as necrosis, expression of CK19 and loss of PR expression are important factors for predicting the malignant behavior and prognosis [15]. Several studies have suggested that patients with elevated serum AFP levels have a poor prognosis, and liver metastases were noted in most of these patients [6-9]. In the present case, serum AFP levels were used as an indicator of therapeutic effectiveness and a marker for monitoring early recurrence and metastasis. The serum AFP levels apparently correlated with the tumor burden and increasing AFP levels might give a warning for timely interventions.

## Conclusion

We described the first case of pure AFP-producing pNEC. Serum AFP levels may provide useful information for monitoring the therapeutic effectiveness, early recurrence or metastases.

## Consent

Written informed consent was obtained from the patient for publication of this Case report and any accompanying images. A copy of the written consent is available for review by the Editor of this journal.

## Abbreviations

AFP: Alpha-fetoprotein
NETs: Neuroendocrine tumors
pNETs: Neuroendocrine tumors of pancreas
HCC: Hepatocellular carcinoma
CT: Computed tomography
CEA: Carcinoembryonic antigen
19–9: Carbohydrate antigen
HCG: Human chorionic gonadotrophin
HPF: High-power fields
CgA: Chromogranin A
Syn: Synaptophysin
CKpan: Pan-cytokeratin
PR: Progesterone receptor
SS: Somatostatin
RFA: Radiofrequency ablation
NECs: Neuroendocrine carcinomas
